# Examining inpatient chemotherapy utilization among patients with cancer and impact on outcomes

**DOI:** 10.1093/oncolo/oyaf214

**Published:** 2025-10-30

**Authors:** Giulia Petrone, Shivani Handa, Justine Anderson, Nobel Chowdhury, Deukwoo Kwon, Priya Jain, Aarti S Bhardwaj, Cardinale B Smith, Natalie S Berger

**Affiliations:** Division of Oncology, Department of Medicine, Washington University School of Medicine, St. Louis, MO 63110, United States; Division of Hematology, Department of Medicine, The Ohio State University Wexner Medical Center, Columbus, OH 43210, United States; Division of Hematology and Medical Oncology, Department of Internal Medicine, Icahn School of Medicine at Mount Sinai, Tisch Cancer Institute, New York, NY 10029, United States; Department of Hematology Oncology, Zucker School of Medicine, Northwell Health, Hempstead, NY 11549, United States; Department of Population Health Science and Policy, Icahn School of Medicine, New York, NY 10029, United States; Division of Clinical and Translational Sciences, Department of Internal Medicine, University of Texas Health Science Center at Houston, Houston, TX 77030, United States; Division of Hematology and Medical Oncology, Department of Internal Medicine, Icahn School of Medicine at Mount Sinai, Tisch Cancer Institute, New York, NY 10029, United States; Division of Hematology and Medical Oncology, Department of Internal Medicine, Icahn School of Medicine at Mount Sinai, Tisch Cancer Institute, New York, NY 10029, United States; Department of Medicine, Memorial Sloan Kettering Cancer Center, New York, NY, United States; Division of Hematology/Oncology, Department of Internal Medicine, Columbia University Irving Medical Center/New York Presbyterian-Hudson Valley Hospital, Cortlandt Manor, NY 10567, United States

**Keywords:** aggressive end-of-life care, inpatient systemic therapy, inpatient immunotherapy, palliative care, quality of life

## Abstract

**Background:**

With the adoption of safer outpatient cancer care practices, much of cancer care has transitioned to outpatient settings, decreasing the need for inpatient systemic therapy (IST), which is associated with poorer end-of-life outcomes. We evaluated reasons for IST use, palliative care (PC) utilization, and outcomes among IST recipients to inform guidelines on appropriate IST use.

**Materials and methods:**

We conducted a retrospective chart review of all IST admissions at an academic center from January 2016 to December 2017. Patients were stratified by solid tumor (ST) vs hematological malignancies (HM). We recorded IST urgency, response, mortality, and other variables. Descriptive statistics and odds ratios were estimated from logistic regression models with mixed effects to account for multiple admissions per patient.

**Results:**

We analyzed 893 admissions (19% ST) among 620 patients. HM patients required frequent elective IST admissions than ST (*P* < .0001). ST patients more often received IST for non-urgent indications (*P* = .0032) during non-cancer-related admissions. ST patients had fewer responses to IST compared to HM (36% vs 70%; *P* < .0001). PC services were more likely utilized for ST vs HM patients (48% vs 14%; *P* < .0001) and were associated with increased rates of health care proxy assignment, code status change, and hospice discharge. Early 60-day mortality was higher for ST vs HM patients (17.3% vs 5.8%; *P* < .001), and most patients (55%) died inpatient during the index admission.

**Conclusion:**

IST was overutilized in ST patients with poor response rates and significant early mortality. PC service utilization rates remain low but improved end-of-life transition planning.

Implications for Practice This study is among the first to describe inpatient systemic therapy (IST) utilization patterns, focusing on the indications and urgency of administration and correlating these with patient outcomes. Our findings highlight opportunities to improve oncology practice by reducing inappropriate IST use, identifying patients unlikely to benefit from aggressive treatment, and underscoring the need for better integration of palliative care. This work lays the foundation for future quality improvement initiatives, such as systemic therapy stewardship programs, that can refine treatment frameworks and reduce aggressive end-of-life interventions.

## Introduction

Advances in cancer care have shifted much of systemic therapy to outpatient settings, offering patients greater convenience and improved quality of life. This transition toward ambulatory cancer care is driven by several factors, including advancements in outpatient treatment modalities, the increasing recognition of the importance of patient-centered care, and the desire to reduce healthcare costs. The treatment of cancer is, however, multifaceted and varies significantly depending on the type—solid tumors (ST) vs hematological malignancies (HM)—and stage of the malignancy. Although the outpatient setting is favored, inpatient administration of systemic treatment continues to be preferred in many instances for HM, such as acute leukemia and high-grade lymphoma, curative intent regimens for germ cell tumors and certain head and neck cancers, imminently life-threatening complications in ST, allogeneic transplants, and chimeric antigen receptor (CAR) T cell therapies. Understanding the clinical factors that drive the inpatient administration of systemic cancer treatment in situations where inpatient systemic therapy (IST) is not the preferred setting is crucial for optimizing patient care and resource allocation within healthcare systems.

Palliative systemic treatment is sometimes given inpatient to reduce symptom burden and prolong life in patients with unresectable metastatic disease. However, studies have shown poor outcomes when administering IST at the end of life.^1–8^ One retrospective review reported inpatient palliative chemotherapy to be associated with aggressive end-of-life care, increased ICU admissions, and high 60-day mortality.^2^ Several other retrospective studies have shown worse outcomes for inpatients who received immunotherapy, including high mortality rates within 30-90 days.^3–5^ Moreover, the rising cost of cancer care, driven in part by inpatient treatments, necessitates a focus on value-based care and reducing unnecessary hospitalizations.^9^^,^^10^ According to the National Cancer Institute, the economic burden associated with cancer-related care was $21 billion in 2019. Patient out-of-pocket costs accounted for $16.22 billion, and patient time cost was $4.87 billion.^11^ Hospitals are reimbursed based on an admission diagnosis code, and often times IST is not reimbursed. Thus, it is imperative we develop sound strategies to reduce inpatient administration without compromising high-quality care and patient outcomes. Chemotherapy delivery in the outpatient setting is beneficial to both patients and the healthcare system as it decreases costs, improves logistical considerations, and improves quality of life. Avoiding unnecessary inpatient stays improves quality care metrics of hospitals by reducing adverse events (hospital-acquired infection), improving patient access (by increasing bed availability), and enhancing patient satisfaction. Outpatient chemotherapy administration also affords patients access to financial assistance programs.^12^ The ASCO Value Framework guides physicians to weigh the side effects, clinical benefits, and symptomatic or quality-of-life improvement from new cancer therapies and assess their value in the context of cost.^13^ This facilitates shared decision-making by oncologists and patients in selecting a high-value treatment in a personalized manner.

At present, there is a lack of clinical data in the United States indicating the current utilization rate of IST and its outcomes. This study aims to identify patients who are likely to benefit from IST by delineating the clinical drivers of IST. We directly compare HM patients, where IST is often required, and ST patients, where outpatient systemic therapy is the standard of care. Through exploring this data, our hope is to create guidelines and protocols to guide decision-making for IST, deliver high-quality care, and augment healthcare quality metrics. We also evaluate the utilization of palliative care services and their impact among patients receiving IST.

## Materials and methods

We conducted a retrospective review of the electronic health record to identify patients with IST administration at a large metropolitan NCI-designated cancer center between January 2016 and December 2017. At the time of our review, our center’s policy required approval from a designated clinical leader for any non-formulary anti-cancer therapy, including immunotherapy, antibody drug conjugates, and other targeted therapies. This process involved submitting a formal email request detailing the patient’s case and the rationale for inpatient administration.

Patients were stratified by ST vs HM. We collected sociodemographic and clinical characteristics at the time of IST administration, including age, sex, race, ethnicity, insurance type, cancer type, cancer stage, Karnofsky Performance Scale (KPS) at the time of index admission, lines of prior therapy (defined as first line or second line and above), and number of emergency department visits as well as hospital admissions before the index one. KPS was preferred over Eastern Cooperative Oncology Group Performance (ECOG) to reflect the practice in our institution.

We collected the reason for admission and identified 4 categories: (1) elective for IST; (2) symptoms directly or indirectly related to the primary cancer (eg, pain) or progression of disease (POD); (3) complications related to cancer-directed therapy (ie, infections, immune-related adverse events); and (4) surgical procedure. Indication for IST administration was assessed and stratified into 6 groups: (1) chemotherapy regimens that are usually administered inpatient such as high-risk ST groups (eg, newly diagnosed small cell lung cancer, head and neck cancer, germ cell tumors, etc.), consolidation regimens for leukemia and lymphoma, conditioning regimens for bone marrow transplant, desensitization protocols, defined as “admitted for IST”; (2) induction regimens for acute leukemia and lymphoma; (3) unplanned due to POD diagnosed during the index admission, defined as “POD”; (4) palliation of cancer-related symptoms (eg, pain) defined as “symptomatic from disease”; (5) patients admitted for non-cancer indications during a planned outpatient treatment cycle who received IST; and (6) the few cases without documentation identifying the reason for IST were classified as “unclear.”

We then classified the reason for IST based on urgency. IST administered for patients “admitted for IST” and patients with acute leukemia or lymphoma requiring induction was always considered urgent, while treatment received because it corresponded to a planned outpatient cycle was always considered non-urgent. In all other admissions, urgency was evaluated on a case-by-case basis, considering the severity of cancer-related symptoms and the clinical implications of POD. The types of treatment comprised “classic chemotherapy,” which included proteasome inhibitors; “immunotherapy,” such as PD-1 and CTLA-4 inhibitors; and “targeted therapy,” including “monoclonal antibodies.” We excluded patients receiving investigational treatments on a clinical trial, including CAR T-cell, as they were not standard of care at the time of our review. We included patients admitted for a bone marrow transplant and classified them as receiving “classic chemotherapy.”

To determine whether patients responded to IST, we reviewed the first available outpatient oncology progress notes following index admission, along with the next available restaging pathology or radiology report. Response assessment was based on the reviewer’s analysis of chart data, including the oncologist’s documentation from the post-hospitalization outpatient visit. Initial chart reviews were conducted by residents and fellows and subsequently verified by a board-certified oncologist. Assessment for response was not based on a prespecified timeline, as interval assessments differ for each malignancy. Response in ST was defined as stable, partial, or complete on imaging after the index admission. Although RECIST provides a more objective measure of ST response, it is often not documented^14^; therefore, response assessment in this study was based on the treating physician’s assessment as documented in clinical notes. In HM, where pathologic assessment is required, bone marrow biopsy results or disease-specific laboratory values were reviewed.

We also evaluated the occurrence and date of important clinical events, including inpatient palliative care consultation (PCC), change of code status to “Do Not Resuscitate” (DNR) and assignment of a health care proxy (HCP). We collected other variables which can impact patient care such as length of stay (LOS) categorized as ≤7 days, 8-14 days, 15-30 days, and >30 days, with a cut-off of >7 days classified as a longer LOS, location of death (inpatient, home, hospice), and 60-day mortality.

This study was determined to be exempt by the Icahn School of Medicine at Mount Sinai Institutional Review Board.

### Statistical analysis 

Descriptive statistics were conducted on baseline characteristics. Continuous variables were summarized by the mean and standard deviation or the median and interquartile range as appropriate, while categorical variables were summarized by frequency (*N*) and percentage (%). Continuous variables were compared between groups using *t*-test or Wilcoxon test, and categorical variables were compared using chi-squared test or Fisher test. Comparison in the reason for admission, urgency of IST and types of IST on patients with ST versus HM were conducted using generalized linear mixed model. Odds ratios (OR) were estimated from logistic regression models with mixed effects to account for correlations from multiple admissions per patient. Cumulative incidence plot and Cox proportional hazard regression models were used to assess the association between mortality and study covariates. All statistical analyses were conducted using SAS (Version 9.4. *SAS Institute* Inc., Cary). All tests were 2-sided, and statistical significance was considered when *P* < .05.

## Results

### Baseline characteristics

We identified 893 admissions where IST was administered between January 2016 and December 2017 to 620 unique patients (Table 1). More patients were admitted with HM (504; 81%) compared to ST (116; 19%; *P* = <.0001). Among HM, multiple myeloma (53%) was the most common, followed by leukemia (26%) and lymphoma (21%). Among ST, gastrointestinal (27%), head and neck (18%), and lung cancers (12%) were most common. When compared with HM, ST patients had a lower functional status with KPS ≤50% in 37% of ST vs 7% of HM patients (*P* < .0001), less lines of systemic treatment prior to index admission (*P* < .0001), and more frequent emergency department visits before the index admission (*P* < .0001; Tables S1 and S2).

**Table 1.** Baseline patient characteristics.

**Variable**	**All patients *N* (%)**	**Hematologic malignancy *N* (%)**	**Solid tumor *N* (%)**	*P*-value^a^
Patients	620 (100)	504	116	<.0001
Age at first admission (median)	60 (19-94)	60 (19-94)	59 (20-90)	.148^b^
Race/Ethnicity				.012
Black	116 (19)	92 (18)	24 (21)	
Hispanic	58 (9)	44 (9)	14 (12)	
Asian/Other/Unknown	187 (30)	166 (33)	21 (18)	
White	259 (42)	202 (40)	57 (49)	
Sex				.918
Female	281 (45)	229 (45)	52 (45)	
Male	339 (55)	275 (55)	64 (55)	
Insurance				.022
Commercial	334 (54)	272 (54)	62 (53)	
Medicare	186 (30)	158 (31)	28 (24)	
Medicaid	69 (11)	55 (11)	12 (12)	
Self-pay/Uninsured	30 (5)	18 (4)	12 (10)	
Unknown	1 (0.2)	1 (0.2)	0	
Tumor type				
Solid malignancy	116 (19)		-	
*GI*	31 (5)	-	31 (27)	
Head and neck	21 (3)	-	21 (18)	
Lung	15 (2)	-	15 (12)	
GYN	14 (2)	-	14 (12)	
GU	13 (2)	-	13 (11)	
Sarcoma	10 (2)	-	10 (9)	
Breast	9 (1)	-	9 (8)	
CNS primary	4 (1)	-	4 (3)	
Hematologic malignancy	504 (81)		-	
Multiple myeloma	267 (43)	267 (53)	-	
Leukemia	131 (21)	131 (26)	-	
Lymphoma	105 (17)	105 (21)	-	
Functional status				<.0001
Karnofsky 80%-100%	244 (76)	218 (43)	26 (22)	
Karnofsky 60%-70%	97 (30)	86 (17)	11 (9)	
Karnofsky ≤50%	77 (24)	34 (7)	43 (37)	
Unknown	202 (63)	166 (33)	36 (31)	

a Denotes *P*-value from Fisher’s exact test.

b Denotes *P*-value from Wilcoxon test.

Abbreviations: CNS = central nervous system primary cancer; GI = gastrointestinal cancer; GU = genitourinary cancer; GYN = gynecological cancer (ovarian, uterine, and cervical); *N* = number.

### Reason for admission, IST administration, type of therapy, and outcomes

When we analyzed the reason for admission, we found that 82% of HM required admission primarily to receive IST as compared to 39% of ST (*P* < .0001; Table 2). ST patients were more frequently admitted for reasons other than chemotherapy, including cancer-related symptoms, cancer complications, and POD (*P* = .003). To assess whether there was an appropriate use of IST, we reviewed the chart and analyzed the indication for treatment. Thirty-five percent of ST admissions were for IST compared to a majority of HM patients (79%). In 22% of ST admissions, patients received IST because the admission coincided with a non-urgent planned cycle compared to only 3% of HM. Other indications for IST were POD (22% of ST, 11% of HM; *P* < .0001) and symptoms from the tumor (18% of ST, 6% of HM; *P* = .051). Overall, ST patients were more likely to receive IST while admitted for unrelated reasons compared to HM (Table 2). We also identified that 41 patients (HM, *n *= 38) required intensive care unit (ICU) stay during the index admission and 9 among them received IST in the ICU. None of the patients received more than one cycle of IST within the same admission.

**Table 2.** Reason for admission, urgency of inpatient systemic treatment (IST), and types of IST.

Variable	All patients *N* (%)	Hematologic malignancy *N* (%)	Solid tumor *N* (%)	***P*-value** ^a^
No of patients	620	504	116	
Line of systemic treatment before index admission				<.0001
≤1 line of systemic treatment	297 (33)	229 (30)	68 (49)	
≥1 lines of systemic treatment	596 (67)	526 (70)	70 (51)	
Reason for admission				<.0001
Elective for IST	673 (75)	616 (82)	57 (39)	<.0001
Symptoms/POD	190 (21)	116 (15)	74 (54)	.003
Cancer related complications	29 (3)	21 (3)	8 (6)	
Surgery	5 (1)	1 (0.1)	4 (3)	
Reason for IST				<.0001
Admitted for IST	553 (62)	504 (67)	49 (35)	<.0001
Induction required for acute leukemia/lymphoma	90 (10)	90 (12)	N/A	
POD	116 (13)	86 (11)	30 (22)	<.0001
Symptomatic from disease	67 (7)	42 (6)	25 (18)	.051
Admitted for unrelated reasons but during planned cycle	54 (6)	24 (3)	30 (22)	.496
Unclear	10 (1)	6 (1)	4 (3)	
Urgency of IST				.010
Urgent	667 (75)	578 (77)	89 (64)	
Not urgent	195 (22)	152 (20)	43 (31)	
Unknown/unclear	31 (3)	25 (3)	6 (4)	
Type of IST				.009
Classic chemo	694 (78)	578 (77)	116 (84)	<.0001
Classic chemo + Target	106 (12)	102 (13)	4 (3)	<.0001
Target therapy	74 (8)	61 (8)	13 (9)	
Classic chemo + IO	6 (1)	5 (1)	1 (1)	
Immunotherapy	12 (1)	8 (1)	4 (3)	
Unknown	1 (0.1)	1 (0.1)	N/A	

a *P*-value from the testing proportion is equal to 50% (ie, HM and ST have equal proportions).

Abbreviations: Chemo = chemotherapy; HM = hematologic malignancy; IO = immunotherapy; IST = inpatient systemic therapy; *N* = number; N/A = not available; POD = progression of disease; ST = solid tumor.

When assessing the indication for IST, we identified that ST patients were significantly more likely to receive IST for a non-urgent indication compared to HM (*P* = .0032; OR 0.54; 95% CI: 0.36-0.82, Table S3). In the adjusted analysis, tumor type was significantly correlated with urgency of IST, with HM patients having a higher association with urgent IST (*P* = .01; OR 0.46; 95% CI: 0.25-0.83). Moreover, the median time from admission to receiving IST was shorter for HM vs ST at 1 day (range, 0-3 days) vs 4 days (range, 1-9 days), respectively (*P* < .0001), indicating a more urgent need to initiate treatment for HM patients.

To better gauge the changing treatment paradigms and ensuing costs, we also analyzed the type of IST administered.^10^ The majority of ST (84%) and HM (77%) patients received classic chemotherapy alone, with HM patients more frequently treated with classic chemotherapy compared to ST (*P* < .0001; Table 2). Immunotherapy and combination of chemo-immunotherapy was infrequently administered (1% HM vs 1%-3% ST). A significantly higher number of HM (13%) received chemotherapy plus targeted therapy compared to ST (3%) patients (*P* < .0001).

Finally, we assessed response to IST (Table 3 and Table S4). Combining both HM and ST admissions, response to any treatment was 65%. Overall, ST patients were significantly less likely to achieve a response to any treatment compared to HM (36% and 70%, respectively; *P* < .0001; OR 0.27; 95% CI: 0.17-0.43). In the adjusted analysis, ST patients had also less likelihood to achieve a response to any treatment compared to HM (*P* < .0001; OR 0.34; 95% CI: 0.20-0.57). Specifically, ST patients were less likely to experience a response than HM patients when treated with classic chemotherapy (*P* < .0001), targeted therapy (*P* = .0006), and chemotherapy plus targeted therapy (*P* < .0001). Additionally, ST and HM patients with KPS ≤50% (*P* < .0001) were less likely to achieve a response to IST. There was no significant difference in response to treatment based on age, race, ethnicity, sex, or stage (Table S4). We subsequently stratified response to IST by prior line of treatment. In the overall cohort, 33% of patients were treatment-naive, while 67% had received one or more lines of treatment (≥1 line) prior to index admission. Among the treatment-naive patients, ST were significantly less likely to respond to IST compared to HM patients (49% vs 83%, respectively; *P* < .0001). Similarly, in the group that had received ≥1 line, ST patients responded in 24% of cases compared to 65% of HM patients (*P* < .0001).

**Table 3.** Distribution of types of inpatient systemic treatment with associated response.

Response	All patients	Hematologic malignancy	Solid tumor	
	*N* (%)	*N* (%)	*N* (%)	*P-*value
Any IST	893 (100)	755 (100)	138 (100)	<.0001
No	281 (31)	216 (29)	65 (47)	
Yes	580 (65)	530 (70)	50 (36)	
Unknown	32 (4)	9 (1)	23 (17)	
Prior lines of therapy	<.0001			
0	297 (33)	229 (30)	68 (49)	<.0001
No	63 (21)	35 (15)	28 (41)	
Yes	222 (75)	189 (83)	33 (49)	
Unknown	12 (4)	5 (2)	7 (10)	
≥1 line	596 (67)	526 (70)	70 (51)	<.0001
No	218 (37)	181 (34)	37 (53)	
Yes	358 (60)	341 (65)	17 (24)	
Unknown	20 (3)	4 (1)	16 (23)	
Classic chemotherapy	694 (100)	578 (100)	116 (100)	<.0001
No	222 (32)	171 (30)	51 (44)	
Yes	446 (64)	400 (69)	46 (40)	
Unknown	26 (4)	7 (1)	19 (16)	
Immunotherapy	12 (100)	8 (100)	4 (100)	.5758^a^
No	7 (58)	4 (50)	3 (75)	
Yes	5 (42)	4 (50)	1 (25)	
Unknown	0	0	0	
Targeted therapy	74 (100)	61 (100)	13 (100)	.0006^a^
No	29 (39)	21 (34)	8 (61)	
Yes	43 (58)	40 (66)	3 (23)	
Unknown	2 (3)	-	2 (15)	
Classic chemo + IO	6 (100)	5 (100)	1 (100)	.3333
No	0	0	0	
Yes	4 (67)	4 (80)	0	
Unknown	2 (33)	1 (20)	1 (100)	
Classic chemo + TT	106 (100)	102 (100)	4 (100)	<.0001
No	22 (21)	19 (19)	3 (75)	
Yes	82 (77)	82 (80)	0	
Unknown	2 (2)	1 (1)	1 (25)	
IO + TT	1 (100)	1 (100)	0	NE
No	1 (100)	1 (100)	0	
Yes	0	0	0	
Unknown	0	0	0	

a Denotes *P*-value from binomial exact test. All other *P*-values were obtained from chi-square test.

Abbreviations: Chemo = chemotherapy (includes traditional cytotoxic drugs: alkylating agents, platinum-based agents, antimetabolites, proteasome inhibitors, such as bortezomib, carfilzomib, etc.); IO = immunotherapy (includes PD-1 inhibitors, PD-L1 inhibitors, CTLA-4 inhibitors, such as pembrolizumab, nivolumab, and atezolizumab); IST = inpatient systemic therapy; *N* = number; NE = not estimable; TT = targeted therapy (includes targeted small molecules, monoclonal antibodies and antibody drug conjugates, such as imatinib, trastuzumab, rituximab, daratumumab, bevacizumab, and cetuximab).

### Palliative care utilization

Inpatient PCC was more likely to be utilized for ST vs HM (*P* < .001; OR 4.35; 95% CI: 2.48-7.65, Table S5). Median time from admission to PCC was significantly shorter for ST at 2 days (range, 0-38 days) vs 8 days (range, 0-50 days) for HM; *P* = .008. Following PCC, a change in status from full code to DNR/DNI was documented in 19.2% and 23.4% HM and ST admissions, respectively, as compared to only 1.6% of HM admissions and 0 ST admissions where PCC was not obtained (*P* < .0001, Table 4). PCC was also associated with a significantly increased likelihood of HCP assignment for both ST (*P* = .001; OR 24.8; 95% CI 3.47-177.4) and HM patients (*P* < .0001; OR 5.86; 95% CI: 3.35-10.3) as well as discharge to hospice in the entire cohort (*P* < .0001; OR 10.7; 95% CI: 4.5-25.6). Finally, lower PS was significantly associated with increased utilization of PCC, with higher rates observed among patients with KPS of 60%-70% (*P* = .0009) and ≤50% (*P* < .0001). Among patients with early mortality (within 60 days of IST administration), PCC was utilized in most of ST patients (90%) as compared to only 54% of HM patients (Table 5).

**Table 4.** Inpatient palliative care utilization and outcomes.

**Variable**	**All admissions** ***N* (%)**	**Hematologic malignancy ** ***N* (%)**	**Solid tumor** ***N* (%)**	***P*-value**
Palliative care consult				<.0001
Consulted	178 (20)	110 (14)	68 (48)	
Not consulted	715 (80)	648 (85)	73 (52)	
Reason for pall care consult				.001
Non pain symptoms (eg, support, depression)	19 (11)	14 (13)	5 (7)	.066
Non pain symptoms + Plan of care	3 (2)	2 (2)	1(1)	
Pain	105 (59)	72 (65)	33 (48)	.0002
Pain + Non pain symptoms	4 (2)	4 (4)	-	
Pain + Plan of care	12 (7)	4 (4)	8 (12)	.387
Plan of care/GOC	25 (14)	13 (12)	12 (18)	1.0
Trigger consult	10 (6)	1 (1)	9(13)	.027
Code status at admission				.756
DNR/DNI	27 (3)	23 (3)	4 (3)	
Full	863 (97)	729 (97)	134 (97)	
Unknown	3 (0.3)	3 (0.4)	-	
Code status change at discharge				<.0001
Full code to DNR/DNI (palliative care consulted)	35/168 (21)	20/104 (19)	15/64 (23)	
Full code to DNR/DNI (palliative care NOT consulted)	10/694 (1)	10/624 (2)	0/70 (0)	
Unknown	4 (0.4)	4 (0.5)	-	
HCP prior to admission				.005
No	516 (58)	419 (55)	97 (70)	
Yes	356 (40)	315 (42)	41 (29)	
Unknown	21 (2)	21 (3)	-	
HCP assigned during admission (palliative care consulted)	84/178 (47)	63/110 (57)	21/68 (31)	<.001 (for HM)^a^ <.001 (for ST)^a^
Patient discharged from index admission				.391
No	35 (4)	27 (4)	8 (6)	
Yes	856 (96)	726 (96)	130 (94)	
Unknown	2 (0.2)	2 (0.3)	-	
Discharge to hospice				<.0001
Palliative care consulted	18/178 (10)	8/103 (8)	10/67 (15)	
Palliative care NOT consulted	7/715 (1)	7/638 (1)	0/70 (0)	

a Reference group.

Abbreviations: DNR = do not resuscitate; DNI = do not intubate; GOC = goals of care; HCP = health care proxy; N/A = not available; *N* = number; pall care = palliative care; SAR = sub-acute rehabilitation.

**Table 5.** Patients with 60-day mortality: characteristics and palliative care utilization.

**Variable**	***N* (%)**	**Solid tumor**	**Hematological malignancy**
All	56	19 (34%)	37 (66%)
Race/Ethnicity			
White	29 (52)	10 (53)	19 (51)
Black	10 (18)	7 (37)	3 (8)
Hispanic	6 (11)	1 (5)	5 (14)
Asian/Other/Unknown	11 (19)	1 (5)	10 (27)
Sex			
Male	35 (63)	11 (58)	24 (65)
Female	21 (37)	8 (42)	13 (35)
Length of stay			
≤7 days	8 (14)	3 (16)	5 (14)
8-14 days	11 (20)	7 (37)	4 (11)
15-30 days	17 (30)	4 (21)	13 (35)
>30 days	15 (27)	5 (25)	10 (27)
Unknown	5 (9)	0 (0)	5 (13)
Consult to palliative care			
Consulted	37 (66)	17 (90)	20 (54)
Not consulted	19 (34)	2 (10)	17 (46)
GOC discussion			
No	21 (38)	10 (53)	11 (30)
Yes	34 (60)	9 (47)	25 (67)
Unknown	1 (2)	0 (0)	1 (3)
Code status at admission			
DNR/DNI	4 (7)	0 (0)	4 (11)
Full	52 (93)	19 (100)	33 (89)
Functional status at the time of inpatient chemotherapy			
Karnofsky: 80%-100%	12 (21)	1 (5)	11 (30)
Karnofsky: 60%-70%	8 (14)	3 (16)	5 (14)
Karnofsky: <=50%	21 (38)	11 (58)	10 (26)
Unknown	15 (27)	4 (21)	11 (30)
Place of death			
Hospice (inpatient and outpatient)	22 (40)	7 (37)	16 (43)
Inpatient	31 (54)	11 (58)	19 (51)
Outpatient	3 (4)	1 (5)	2 (6)

Abbreviations: DNI = do not intubate; DNR = do not resuscitate; GOC = goals of care.

### Length of stay

Patients with ST had a significantly shorter median LOS at 6 days (0-71 days) compared to HM at 15 days (1-171 days) (*P* < .0001; OR 9.71; 95% CI: 3.51-26.84; Table S6). There was no difference in the proportion of patients who had a prolonged LOS among those with HM and ST (16% vs 16.5%). Only tumor type (ST; *P* < .0001) and functional status impacted LOS, with patients having a KPS ≤50% (*P* = .0005) being more likely to have a prolonged inpatient stay (Table S6).

### Mortality

**Figure 1.**
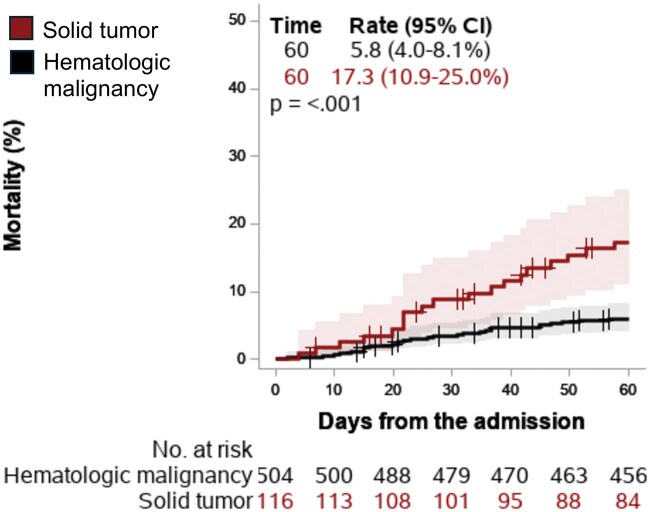
Kaplan–Meier curve for 60-day mortality from index admission. 60-day mortality from index admission in patients with hematologic malignancy and solid tumor.

We evaluated 60-day mortality from the index admission and characterized the association between the collected variables and mortality. With a median follow-up of 48 months for HM and 15.8 months for ST, median OS was 22.2 months for ST and not reached for HM patients. A total of 56 (9%) patients who received IST died within 60 days of admission. Fifty-two out of 56 were full code on admission. On Cox proportional hazard regression analysis (Table S7), risk of death was higher in those aged >65 (HR = 1.46; *P* = .033), insured with Medicaid (HR = 1.58, *P* = .034), admitted for uncontrolled symptoms/complications related to their disease or with POD (HR = 1.80; *P* = .003), and having a longer length of stay >30 days (HR = 1.68, *P* = .040). Moreover, the 60-day mortality rate (Figure 1) was significantly higher for ST vs HM patients (17.3% vs 5.8%, *P* < .001). As expected, patients that achieved a response to IST were less likely to die within 60 days (*P* < .0001; HR = 0.2; 95% CI: 0.14-0.27; Table S7). However, there was no relation between the type of IST and early mortality.

For patients who died within 60 days of admission, PCC was obtained in 66% of cases, and goals of care discussions (GOC) were documented in 60.7% (Table 5). Thirty (55.4%) patients died during index admission, and 23 (41%) were under hospice care at the time of death. Of the 9 patients who received IST during ICU admission, three died within 60 days of receiving IST.

## Discussion

We analyzed 893 hospital admissions, highlighting disparities in treatment urgency, effectiveness, and outcomes between HM and ST patients receiving inpatient chemotherapy. HM patients were more likely to receive IST for more appropriate and urgent indications with better response rates compared to ST patients, who often had worse functional status, received IST for non-­urgent indications, more frequently required a PCC for cancer-­related symptoms, and experienced a significantly higher early mortality.

ST patients had worse documented performance status (PS) compared to HM patients, with over a third of ST patients having a KPS of ≤50%. In our review of the literature, there are no prior data directly comparing PS of HM versus ST patients receiving IST. The discrepancy in PS is likely due to the different reasons for admission between these groups. In fact, HM patients often required urgent direct admission for IST as compared to ST patients, reflecting current standard treatment practice for HM.^15^ Although there have been recent studies demonstrating the feasibility and benefits of ambulatory administration of high-intensity chemotherapy for leukemia and lymphoma, this has been slow to reach routine clinical practice.^16–19^ In contrast, ST patients were frequently admitted for disease complications or POD, consistent with prior studies showing higher ED visits and inpatient admissions for ST patients due to cancer-related complications.^20^^,^^21^

Most ST patients received IST for palliative purposes, particularly for symptom management or POD, with a smaller number receiving their scheduled outpatient regimens during hospitalization. This is consistent with literature indicating that ST patients often receive palliative IST when admitted for cancer-related complications.^2^^,^^22^ The difference in IST indication, predominantly palliative in ST versus curative in HM patients, also explains why ST patients had lower response rates to IST compared to HM patients, regardless of type of IST or number of prior lines of treatment before index admission.^23^ The high prevalence of patients with KPS ≤50% in our ST group is a significant predictor of reduced treatment response.^2^ Additionally, cancer-related symptoms at the time of palliative chemotherapy have been linked to decreased treatment response and hastened death.^24^

Early palliative care introduction has been shown to benefit patients with advanced cancer.^25–27^ The reasons as to why vary, but the common thread is that patients who utilize palliative care services are more likely to choose to continue care in line with their values, cultures, and beliefs. Of the 893 admissions analyzed, 19.9% used palliative care services, indicating underutilization despite its known benefits.^28^ ST patients and patients with KPS ≤50% were more likely to receive PCC, most commonly for pain and GOC conversations.^29–31^ Inpatient PCC was associated with higher rates of discharge to hospice and changes in code status from full code at admission to DNR/DNI reflecting the importance of palliative care intervention while also underscoring the need for better prognostication and earlier introduction of PCC in the outpatient setting.^28^^,^^32^^,^^33^

We observed significantly higher early (60-day) mortality in ST patients (17.3%) vs HM (5.8%), underscoring the limited benefit of palliative IST in ST. A group from Brazil focusing on patients with poor PS (ECOG 3-4) reported a much higher rate of ∼60% 60-day mortality.^2^ In our cohort, 60-day mortality was also higher (27%) among patients with KPS ≤50% which resonates with a large retrospective study showing that ECOG >2 was associated with increased risk of 90-day mortality in patients receiving palliative chemotherapy.^34^ Most deaths (55%) occurred within the same admission and in patients who were full code (92%), underscoring the need for better palliative care integration and prognostic awareness.

We also compared LOS between HM and ST patients. As expected, LOS was longer for HM patients, reflecting the complexity of inpatient management, especially if requiring induction chemotherapy or stem cell transplant. In contrast, ST patients had an average shorter LOS by 9 days because they typically receive single-agent chemotherapy regimens, which are administered over a few hours or less and would not require an inpatient stay.

It is evident that the patients who make up these two groups are different in terms of what brought them to the hospital, the urgency with which they need treatment, and their overall PS. IST for HM patients is often administered in a coordinated fashion per standardized institutional protocols, whereas ST patients are often admitted for an expected or unexpected complication related to treatment or disease. The decision to offer cancer-directed therapy as an inpatient can be highly subjective for ST patients, which makes it challenging to assess its appropriateness and impact on overall outcomes. ST and HM groups often utilize IST for different indications and therefore should be thought about and evaluated accordingly. IST is associated with lower response rates and higher mortality in patients with advanced-stage ST, which was not observed to the same extent in the HM group. To our knowledge, this is the first study to examine treatment practices across different cancer types and disease states. These data, at a time when utilization data are lacking, provide insight into not only treatment at our institution but also standard oncology practice. Two novel opportunities exist because of this study. The first is the opportunity to change practice around non-urgent admissions for IST, and the second is to address aggressive end-of-life care. From this study, we can examine our delivery of care to these two different patient populations and evaluate the benefit of IST.

### Future directions

Our findings serve as a launchpad into further quality improvement initiatives, with the main initiative being the development of a systemic therapy stewardship program and process to decrease inappropriate use of IST. Based off our study, Bhardwaj et al.^33^ implemented a pilot program to reduce non-formulary IST administration by using an automated scoring rubric and secondary committee to review each IST request. Taking it a step further, our institution is examining our treatment framework and opportunity to move chemotherapy administration to the outpatient setting and even at home, as other programs have.^17^^,^^33^ While our study looked at all admissions where IST was administered, future studies should examine the outcomes and PCC rates among patients who receive IST specifically during an unplanned admission to further clarify the utility of this practice. It is also important to gather data on patient perspectives on aggressive end-of-life care to design a framework for value-based care.

### Limitations

Our study has several limitations. First, it was conducted at a single urban academic medical center, limiting generalizability. Second, as a retrospective cross-sectional study, it has inherent selection bias, and third, although groups were selected based on tumor type, this does not assure generalizability.

While our study reviewed IST use from 2016 to 2017, many of the treatment practices described remain standard today. Over the past decade, while clinical practices have evolved, many of the backbone chemotherapy agents have not undergone substantial modifications.^35–37^ Our study encompassed many of the currently used immunotherapies and targeted therapies—such as PD-1 and PD-L1 inhibitors, monoclonal antibodies, and tyrosine kinase inhibitors—as they were already standard of care during our review period. Although new targeted therapies have since emerged, their high costs often restrict their use to outpatient settings. Assessing the impact of IST on patient outcomes is still an essential area of research, and our study is among the first to address this topic.

## Conclusion

This study helps to address one of the common situations oncologists frequently encounter—does this patient need cancer-directed therapy while hospitalized? For some of our patients, the answer was a clear yes, and for others there was no added benefit. In fact, for many, administration of IST was consistent with aggressive end-of-life care and a significant 60-day mortality. Our role as physicians is to weigh the risks and benefits of the treatments we deliver to our patients. Through shared decision-making, we must consider the impact on quality of life, patients’ goals, financial burdens associated with the therapies delivered, and truly consider if there is a downside to waiting until after hospital discharge. Our study highlights that IST is frequently overutilized with unclear benefits, and it warrants additional studies on how best to tailor this practice.

## Supplementary Material

oyaf214_Supplementary_Data

## Data Availability

The data underlying this article are available in the article and in its online supplementary material.
