# Supplementary material for: Examining inpatient chemotherapy utilization among patients with cancer and impact on outcomes
Source: Oncologist. 2025 Oct 30;30(11):oyaf214. doi: 10.1093/oncolo/oyaf214 (PMC12640121; doi:10.1093/oncolo/oyaf214)
Supplement: oyaf214_Supplementary_Data [file oyaf214_supplementary_data.zip › The Oncologist_Supplement_resubmission.docx]

**Table S1. Emergency department visits before index admission**

From Zero-inflated Negative Binomial regression model, RR for ST vs HM (ref)=2.40 (95% CI=1.64 to 3.51); p<0.0001.

ST group has 140% higher emergency department visits before IST compared to HM.

|  | **Emergency department visits** | | | | | | | | | | | | |
| --- | --- | --- | --- | --- | --- | --- | --- | --- | --- | --- | --- | --- | --- |
|  | 0 | 1 | 2 | 3 | 4 | 5 | 6 | 7 | 8 | 10 | 12 | 17 | Total |
| HM (N=504) | 315 | 76 | 29 | 11 | 6 | 5 | 1 | 1 | 1 | 1 | 0 | 0 | 446 |
| ST (N=116) | 61 | 26 | 7 | 5 | 5 | 5 | 4 | 0 | 0 | 0 | 1 | 1 | 115 |
| Total | 376 | 102 | 36 | 16 | 11 | 10 | 5 | 1 | 1 | 1 | 1 | 1 | 561 |

**Table S2. Hospital admissions before index admission**

From Negative Binomial regression model, RR for ST vs HM (ref)=1.14 (95% CI=0.87 to 1.49); p=0.354.

ST group does not have higher admissions before IST compared to HM.

|  | **Hospital admissions** | | | | | | | | | | | | | | | | | | |
| --- | --- | --- | --- | --- | --- | --- | --- | --- | --- | --- | --- | --- | --- | --- | --- | --- | --- | --- | --- |
|  | 0 | 1 | 2 | 3 | 4 | 5 | 6 | 7 | 8 | 9 | 10 | 11 | 12 | 13 | 14 | 15 | 23 | 27 | Total |
| HM (N=504) | 171 | 102 | 58 | 41 | 22 | 15 | 10 | 12 | 6 | 2 | 5 | 1 | 1 | 3 | 0 | 1 | 1 | 1 | 452 |
| ST (N=116) | 33 | 29 | 21 | 9 | 8 | 4 | 2 | 3 | 1 | 0 | 2 | 2 | 0 | 1 | 1 | 0 | 0 | 0 | 116 |
| Total | 204 | 131 | 79 | 50 | 30 | 19 | 12 | 15 | 7 | 2 | 7 | 3 | 1 | 4 | 1 | 1 | 1 | 1 | 568 |

Table S1 and S2. Legend: CI = confidence interval; ref = reference; HM = hematologic malignancy; IST = inpatient systemic therapy; N = number; RR = relative risk; ST = solid tumor.

**Table S3. Urgency of inpatient systemic therapy (IST): *Univariate and multivariate analysis of urgency for administration of IST.***

| **Variable** | **UVA** |  | **MVA** |  |
| --- | --- | --- | --- | --- |
|  | **OR (95%CI)** | **p-value** | **OR (95%CI)** | **p-value** |
| **Type of malignancy** |  |  |  |  |
| Hematology Malignancy | ref | - | ref | - |
| Solid tumor | 0.54 (0.36, 0.82) | 0.0032 | 0.46 (0.25, 0.83) | 0.0101 |
| **Age** |  |  |  |  |
| ≤65 | ref | - | ref | - |
| >65 | 1.10 (0.77, 1.57) | 0.5936 | 1.18 (0.73, 1.91) | 0.4923 |
| **Race/Ethnicity** |  |  |  |  |
| White | ref | - | ref | - |
| Black | 0.94 (0.60, 1.49) | 0.8059 | 0.83 (0.49, 1.40) | 0.4816 |
| Hispanic | 3.20 (1.42, 7.22) | 0.0050 | 3.11 (1.31, 7.34) | 0.0098 |
| Asian/Other/Unknown | 0.84 (0.58, 1.21) | 0.3527 | 0.94 (0.60, 1.49) | 0.8031 |
| **Sex** |  |  |  |  |
| Male | ref | - | ref | - |
| Female | 1.08 (0.79, 1.49) | 0.6246 | 1.17 (0.80, 1.70) | 0.4162 |
| **Insurance** |  |  |  |  |
| Commercial | ref | - | ref | - |
| Medicaid | 0.82 (0.48, 1.40) | 0.4661 | 0.75 (0.42, 1.36) | 0.3445 |
| Medicare | 0.68 (0.47, 0.97) | 0.0345 | 0.49 (0.30, 0.78) | 0.0025 |
| Self-pay/Uninsured | 0.64 (0.20, 2.05) | 0.4519 | 1.18 (0.31, 4.40) | 0.8092 |
| **Stage** |  |  |  |  |
| Stage I | ref | - | ref | - |
| Stage II | 0.57 (0.23, 1.38) | 0.2111 | 0.55 (0.19, 1.59) | 0.2694 |
| Stage III | 1.28 (0.56, 2.91) | 0.5549 | 1.25 (0.46, 3.36) | 0.6633 |
| Stage IV | 0.79 (0.36, 1.77) | 0.5719 | 0.86 (0.31, 2.32) | 0.7589 |
| Unknown | 0.78 (0.36, 1.69) | 0.5333 | 0.67 (0.26, 1.72) | 0.4031 |
| **Reason for Admission** |  |  |  |  |
| Elective for IST | ref | - | ref | - |
| Cancer related complications | 0.43 (0.20, 0.94) | 0.0341 | 0.31 (0.12, 0.78) | 0.0137 |
| Surgery | 0.28 (0.04, 1.99) | 0.2027 | 0.33 (0.04, 2.73) | 0.3067 |
| Symptoms/POD | 0.96 (0.64, 1.43) | 0.8387 | 1.05 (0.62, 1.79) | 0.8608 |
| **Functional status** |  |  |  |  |
| Karnofsky 80-100% | ref | - | ref | - |
| Karnofsky 60-79% | 0.82 (0.52, 1.29) | 0.3897 | 1.00 (0.61, 1.66) | 0.9923 |
| Karnofsky <=59% | 0.63 (0.38, 1.07) | 0.0850 | 0.84 (0.42, 1.68) | 0.6251 |
| Unknown | 3.04 (1.91, 4.84) | <.0001 | 3.63 (2.18, 6.05) | <.0001 |

Table S3. Legend: CI = confidence interval; IST = inpatient systemic therapy; MVA = multivariate; POD = progression of disease; OR = odds ratio; Ref = reference; UVA = univariate.

**Table S4. Response to inpatient systemic therapy (IST):** ***Univariate and multivariate analysis of likelihood to respond to IST.***

| **Category** | **UVA** | | **MVA** | |
| --- | --- | --- | --- | --- |
|  | **OR (95% CI)** | **p-value** | **OR (95% CI)** | **p-value** |
| **Type of malignancy** |  |  |  |  |
| Solid tumor | 0.27 (0.17, 0.43) | <.0001 | 0.34 (0.20, 0.57) | <.0001 |
| Hematologic malignancy | Ref | . | Ref | . |
| **Age** |  |  |  |  |
| >65 | 1.38 (0.96, 1.97) | 0.0798 | 1.10 (0.74, 1.63) | 0.6501 |
| ≤65 | Ref | . | Ref | . |
| **Race/Ethnicity** |  |  |  |  |
| Black | 0.87 (0.58, 1.32) | 0.5125 | 0.81 (0.51, 1.30) | 0.3859 |
| Hispanic | 1.58 (0.88, 2.83) | 0.1226 | 1.60 (0.87, 2.95) | 0.1288 |
| Asian/Other/Unknown | 1.13 (0.80, 1.61) | 0.4852 | 0.94 (0.64, 1.38) | 0.7591 |
| White | Ref | . | Ref | . |
| **Sex** |  |  |  |  |
| Female | 0.97 (0.73, 1.31) | 0.8623 | 1.04 (0.76, 1.43) | 0.8019 |
| Male | Ref | . | Ref | . |
| **Insurance** |  |  |  |  |
| Medicaid | NE | - | NI | - |
| Medicare | NE | - | NI | - |
| Self-pay / Uninsured | NE | - | NI | - |
| Commercial | Ref | . | Ref | . |
| **Stage** |  |  |  |  |
| Stage II | 0.57 (0.25, 1.33) | 0.1956 | 0.61 (0.24, 1.55) | 0.3013 |
| Stage III | 0.53 (0.25, 1.12) | 0.0950 | 0.55 (0.24, 1.24) | 0.1485 |
| Stage IV | 0.38 (0.18, 0.80) | 0.0115 | 0.67 (0.29, 1.53) | 0.3407 |
| Unknown | 0.64 (0.31, 1.32) | 0.2238 | 0.54 (0.25, 1.18) | 0.1235 |
| Stage I | Ref | . | Ref | . |
| **Functional status** |  |  |  |  |
| Karnofsky: 60-70% | 0.97 (0.61, 1.53) | 0.8799 | 0.92 (0.57, 1.48) | 0.7266 |
| Karnofsky: <=50% | 0.17 (0.10, 0.29) | <.0001 | 0.25 (0.14, 0.44) | <.0001 |
| Unknown | 0.73 (0.52, 1.03) | 0.0694 | 0.79 (0.55, 1.15) | 0.2176 |
| Karnofsky: 80-100% | Ref | . | Ref | . |

Table S4. Legend: CI = confidence interval; MVA = multivariate; NE = not estimable; NI = not included; OR = odds ratio; Ref = reference; UVA = univariate.

**Table S5. Palliative care.** ***Univariate and multivariate analysis of likelihood of utilization of palliative care services***

| **Category** | **UVA** | | **MVA** | |
| --- | --- | --- | --- | --- |
|  | **OR (95% CI)** | **p-value** | **OR (95% CI)** | **p-value** |
| **Type of malignancy** |  |  |  |  |
| Solid tumor | 5.96 (4.01, 8.85) | <.0001 | 4.35 (2.48, 7.65) | <.0001 |
| Hematologic malignancy | Ref | . | Ref | . |
| **Age** |  |  |  |  |
| >65 | 0.68 (0.44, 1.06) | 0.0874 | 1.04 (0.61, 1.78) | 0.8720 |
| ≤65 | Ref | . | Ref | . |
| **Race** |  |  |  |  |
| Black | 1.21 (0.75, 1.94) | 0.4415 | 1.18 (0.65, 2.11) | 0.5902 |
| Hispanic | 1.25 (0.72, 2.18) | 0.4217 | 1.21 (0.58, 2.54) | 0.6096 |
| Asian/Other/Unknown | 0.61 (0.39, 0.94) | 0.0265 | 0.62 (0.35, 1.10) | 0.1003 |
| White | Ref | . | Ref | . |
| **Sex** |  |  |  |  |
| Female | 0.89 (0.64, 1.24) | 0.5059 | 0.67 (0.44, 1.03) | 0.0660 |
| Male | Ref | . | Ref | . |
| **Insurance** |  |  |  |  |
| Medicaid | 1.27 (0.75, 2.16) | 0.3801 | 1.52 (0.79, 2.91) | 0.2091 |
| Medicare | 0.84 (0.55, 1.27) | 0.4035 | 1.01 (0.63, 1.62) | 0.9593 |
| Self-pay / Uninsured | 63.57 (7.52, 537.77) | 0.0001 | 13.25 (1.53, 114.98) | 0.0190 |
| Commercial | Ref | . | Ref | . |
| **Stage** |  |  |  |  |
| Stage II | 3.88 (1.06, 14.16) | 0.0399 | 3.03 (0.67, 13.66) | 0.1489 |
| Stage III | 2.16 (0.61, 7.58) | 0.2316 | 2.00 (0.47, 8.56) | 0.3493 |
| Stage IV | 6.26 (1.87, 20.93) | 0.0029 | 1.90 (0.43, 8.37) | 0.3958 |
| Unknown | 2.59 (0.78, 8.61) | 0.1201 | 3.15 (0.77, 12.96) | 0.1112 |
| Stage I | Ref | . | Ref | . |
| **Functional status** |  |  |  |  |
| Karnofsky: 60-70% | 2.48 (1.45, 4.25) | 0.0009 | 2.61 (1.48, 4.58) | 0.0009 |
| Karnofsky: <=50% | 30.45 (16.42, 56.45) | <.0001 | 19.95 (10.17, 39.12) | <.0001 |
| Unknown | 0.90 (0.54, 1.49) | 0.6767 | 0.78 (0.45, 1.37) | 0.3909 |
| Karnofsky: 80-100% | Ref | . | Ref | . |

Table S5. Legend: CI = confidence interval; MVA = multivariate; OR = odds ratio; Ref = reference; UVA = univariate.

**Table S6. Length of stay (LOS). *Univariate and multivariate analysis of likelihood of LOS <7 days.***

| **Category** | **UVA** | | **MVA** | |
| --- | --- | --- | --- | --- |
|  | **OR (95% CI)** | **p-value** | **OR (95% CI)** | **p-value** |
| **Type of malignancy** |  |  |  |  |
| Solid tumor | 8.25 (4.14, 16.43) | <.0001 | 9.71 (3.51, 26.84) | <.0001 |
| Hematologic malignancy | Ref | . | Ref | . |
| **Age** |  |  |  |  |
| >65 | NE | - | NI | - |
| ≤65 | Ref | . | Ref | . |
| **Race** |  |  |  |  |
| Black | 1.19 (0.49, 2.86) | 0.6996 | 1.63 (0.63, 4.22) | 0.3124 |
| Hispanic | 2.05 (0.77, 5.44) | 0.1489 | 2.38 (0.79, 7.21) | 0.1235 |
| Asian/Other/Unknown | 0.44 (0.15, 1.25) | 0.1244 | 0.63 (0.19, 2.09) | 0.4525 |
| White | Ref | . | Ref | . |
| **Sex** |  |  |  |  |
| Female | 1.23 (0.63, 2.41) | 0.5419 | 1.77 (0.78, 4.01) | 0.1706 |
| Male | Ref | . | Ref | . |
| **Insurance** |  |  |  |  |
| Medicaid | 0.65 (0.22, 1.91) | 0.4372 | 0.45 (0.13, 1.58) | 0.2123 |
| Medicare | 0.54 (0.24, 1.22) | 0.1371 | 0.41 (0.14, 1.16) | 0.0923 |
| Self-pay / Uninsured | 0.93 (0.11, 7.52) | 0.9431 | 1.10 (0.18, 6.70) | 0.9153 |
| Commercial | Ref | . | Ref | . |
| **Stage** |  |  |  |  |
| Stage II | 1.04 (0.10, 11.02) | 0.9759 | 1.99 (0.22, 18.01) | 0.5414 |
| Stage III | 1.65 (0.20, 13.58) | 0.6429 | 2.70 (0.39, 18.53) | 0.3130 |
| Stage IV | 5.75 (0.75, 43.83) | 0.0917 | 6.37 (0.88, 46.05) | 0.0667 |
| Unknown | 0.53 (0.06, 4.72) | 0.5711 | 0.84 (0.10, 6.84) | 0.8744 |
| Stage I | Ref | . | Ref | . |
| **Functional status** |  |  |  |  |
| Karnofsky: 60-70% | 0.62 (0.20, 1.95) | 0.4120 | 0.40 (0.11, 1.48) | 0.1702 |
| Karnofsky: <=50% | 0.45 (0.10, 2.02) | 0.2968 | 0.02 (0.00, 0.19) | 0.0005 |
| Unknown | 1.62 (0.77, 3.42) | 0.2015 | 0.96 (0.39, 2.36) | 0.9287 |
| Karnofsky: 80-100% | Ref | . | Ref | . |

Table S6. Legend: CI = confidence interval; MVA = multivariate; NE = not estimable; NI = not include; OR = odds ratio; Ref = reference; UVA = univariate.

**Table S7: Univariate and multivariate analysis for factors affecting mortality**

| **Category** | **UVA** | | **MVA** | |
| --- | --- | --- | --- | --- |
|  | **HR (95% CI)** | **p-value** | **HR (95% CI)** | **p-value** |
| **Type of malignancy** |  |  |  |  |
| Solid tumor | 2.06 (1.48, 2.86) | <.0001 | 1.09 (0.68, 1.76) | 0.7116 |
| Hematologic malignancy | Ref | . | Ref | . |
| **Age** |  |  |  |  |
| >65 | 1.73 (1.32, 2.27) | <.0001 | 1.46 (1.03, 2.08) | 0.0333 |
| ≤65 | Ref | . | Ref | . |
| **Race** |  |  |  |  |
| Black | 0.88 (0.61, 1.26) | 0.4783 | 0.78 (0.53, 1.16) | 0.2173 |
| Hispanic | 0.67 (0.39, 1.16) | 0.1513 | 0.65 (0.37, 1.17) | 0.1513 |
| Asian/Other/Unknown | 0.80 (0.58, 1.10) | 0.1715 | 0.73 (0.51, 1.03) | 0.0768 |
| White | Ref | . | Ref | . |
| **Sex** |  |  |  |  |
| Female | 0.93 (0.71, 1.21) | 0.5845 | 0.95 (0.71, 1.27) | 0.7129 |
| Male | Ref | . | Ref | . |
| **Insurance** |  |  |  |  |
| Medicaid | 1.79 (1.21, 2.65) | 0.0033 | 1.58 (1.03, 2.42) | 0.0346 |
| Medicare | 1.30 (0.96, 1.76) | 0.0873 | 1.11 (0.77, 1.59) | 0.5901 |
| Self-pay / Uninsured | 5.77 (2.63, 12.65) | <.0001 | 2.19 (0.92, 5.20) | 0.0757 |
| Commercial | Ref | . | Ref | . |
| **Stage** |  |  |  |  |
| Stage II | 2.57 (0.97, 6.82) | 0.0577 | 1.82 (0.66, 5.03) | 0.2486 |
| Stage III | 2.58 (1.04, 6.43) | 0.0414 | 2.06 (0.81, 5.22) | 0.1298 |
| Stage IV | 3.99 (1.59, 10.00) | 0.0031 | 2.37 (0.90, 6.22) | 0.0805 |
| Unknown | 3.20 (1.29, 7.89) | 0.0118 | 3.02 (1.18, 7.72) | 0.0212 |
| Stage I | Ref | . | Ref | . |
| **Functional status** |  |  |  |  |
| Karnofsky: 60-70% | 1.28 (0.86, 1.91) | 0.2293 | 1.33 (0.86, 2.05) | 0.1986 |
| Karnofsky: <=50% | 3.11 (2.09, 4.63) | <.0001 | 1.08 (0.64, 1.82) | 0.7798 |
| Unknown | 1.17 (0.84, 1.63) | 0.3517 | 1.02 (0.71, 1.47) | 0.9172 |
| Karnofsky: 80-100% | Ref | . | Ref | . |
| **Reason for admission** |  |  |  |  |
| Cancer related complications | 1.28 (0.57, 2.91) | 0.5515 | 0.71 (0.28, 1.78) | 0.4669 |
| Surgery | 3.92 (1.45, 10.62) | 0.0072 | 2.76 (0.92, 8.28) | 0.0709 |
| Symptoms/POD | 3.81 (2.87, 5.06) | <.0001 | 1.80 (1.22, 2.66) | 0.0033 |
| Elective for IST | Ref |  | Ref |  |
| **Palliative care** |  |  |  |  |
| Consulted | 3.70 (2.79, 4.92) | <.0001 | 1.56 (1.05, 2.32) | 0.0264 |
| Not consulted | Ref |  | Ref |  |
| **LOS** |  |  |  |  |
| >30 days | 2.13 (1.40, 3.24) | 0.0004 | 1.68 (1.02, 2.76) | 0.0400 |
| 15-30 days | 0.98 (0.66, 1.47) | 0.9391 | 1.15 (0.74, 1.79) | 0.5237 |
| 8-17 dyas | 1.04 (0.67, 1.63) | 0.8580 | 1.16 (0.73, 1.87) | 0.5286 |
| ≤7 days | Ref |  | Ref |  |
| **Response to IST** |  |  |  |  |
| Yes | 0.17 (0.13, 0.22) | <.0001 | 0.20 (0.14, 0.27) | <.0001 |
| No | Ref |  | Ref |  |

Table S7. Legend: CI = confidence interval; IST = inpatient systemic therapy; LOS = length of stay; MVA = multivariate; OR = odds ratio; Ref = reference; UVA = univariate.
